# PlotTwist: A web app for plotting and annotating continuous data

**DOI:** 10.1371/journal.pbio.3000581

**Published:** 2020-01-13

**Authors:** Joachim Goedhart

**Affiliations:** Swammerdam Institute for Life Sciences, Section of Molecular Cytology, van Leeuwenhoek Centre for Advanced Microscopy, University of Amsterdam, Amsterdam, the Netherlands

## Abstract

Experimental data can broadly be divided in discrete or continuous data. Continuous data are obtained from measurements that are performed as a function of another quantitative variable, e.g., time, length, concentration, or wavelength. The results from these types of experiments are often used to generate plots that visualize the measured variable on a continuous, quantitative scale. To simplify state-of-the-art data visualization and annotation of data from such experiments, an open-source tool was created with R/shiny that does not require coding skills to operate it. The freely available web app accepts wide (spreadsheet) and tidy data and offers a range of options to normalize the data. The data from individual objects can be shown in 3 different ways: (1) lines with unique colors, (2) small multiples, and (3) heatmap-style display. Next to this, the mean can be displayed with a 95% confidence interval for the visual comparison of different conditions. Several color-blind-friendly palettes are available to label the data and/or statistics. The plots can be annotated with graphical features and/or text to indicate any perturbations that are relevant. All user-defined settings can be stored for reproducibility of the data visualization. The app is dubbed PlotTwist and runs locally or online: https://huygens.science.uva.nl/PlotTwist

## Abstract 2

This Community Page article describes PlotTwist, an open source app created with R/shiny to simplify state-of-the-art visualization and annotation of results from experiments that generate continuous data; the app runs locally or online, and no coding skills are needed to use it.

## Introduction

It has been stressed over the years that showing the actual data from individual measurements, instead of summaries only, leads to a better understanding and more complete picture of an experimental result [1–3]. This holds true for data acquired under static conditions as well as for time-dependent data to study dynamics [4]. For static, discrete conditions, dotplots are a good way to graphically depict the data. Several online data visualization tools have been reported that include the option to display the actual data [4–8].

Tools for visualizing continuous data have been reported [9,10], but these have a general purpose and lack several features that are often used to show experimental data. A dedicated, online tool for the visualization of continuous data from scientific experiments is (to the best of our knowledge) lacking. This is surprising, because understanding dynamic systems, especially relevant in biology, requires experimentation over time. In addition, time-series experiments can be high content [11] and may include the application of multiple, different perturbations. The complexity of time-series data requires a tool to rapidly inspect the data and have access to different visualizations. Next to that, it is important to clearly communicate the treatments/perturbations that were applied during the experiment. Besides time-lapse experiments, many other experimental strategies deliver continuous data. Examples include measurements over length, concentration, or wavelength. These types of data are often depicted in plots that visualize the measured variable on a quantitative, continuous scale.

Ideally, a data visualization tool for continuous data would be freely available, be open source, and generate high-quality graphs that are publication ready. Having previously reported such a web tool for discrete data [6], and given the enthusiastic response by the community, we were motivated to generate a web tool for continuous data. Although the focus of the web tool is on time-series data, it can be generally used for any data that are continuous.

The new web tool that we generated is dubbed PlotTwist, for plotting data from Time-lapse experiments With Indicators of conditions at Set Times. This free and open-source app is developed to facilitate the rapid visualization and annotation of continuous data. Creating graphs with PlotTwist does not require coding skills, yet it produces the state-of-the-art data visualization offered by the ggplot2 package. Therefore, we think that PlotTwist is well suited to inspect data and generate publication-quality visualizations. The features and use of PlotTwist are highlighted next.

## Availability, code, and issues

The PlotTwist web tool is available at https://huygens.science.uva.nl/PlotTwist or https://goedhart.shinyapps.io/PlotTwist/

The code was written in R using R (https://www.r-project.org) and Rstudio (https://www.rstudio.com). To run the app, several freely available packages are required: shiny, ggplot2, dplyr, tidyr, readr, readxl, magrittr, ggrepel, DT, dtw [12], shinycssloaders, NbClust [13]. The code of the version 1.0.4 that is reported in this manuscript is archived at Zenodo.org: https://doi.org/10.5281/zenodo.3539121.

Up-to-date code and new releases will be made available on Github, together with information on running the app locally: https://github.com/JoachimGoedhart/PlotTwist.

Users are encouraged to report any errors or unexpected behavior of the web tool. The Github page of PlotTwist is the preferred way to communicate issues and request features (https://github.com/JoachimGoedhart/PlotTwist/issues). Alternatively, the users can contact the author by email or via Twitter. Contact information is found on the “About” page of the app.

## Data input, structure, and normalization

The data can be provided by copy/paste or via upload of a comma-separated values (CSV) or eXceL Spreadsheet (XLS) file. A single line header is used to retrieve the names of the columns. When spaces are encountered in the column names, these are converted to an underscore. The data structure can be either a wide, spreadsheet-type format or a long, tidy format. Example data, of which the details can be found in earlier publications [14,15], are included in the app.

### Wide data format

**Fig 1:**
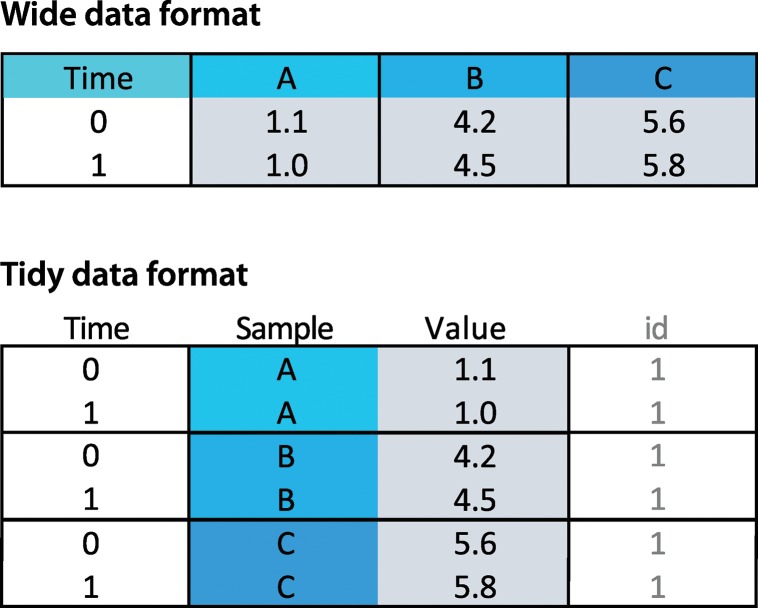
The wide versus tidy data format for time-dependent data. In the wide (spreadsheet-like) data format, the first column holds the time data, and the other columns represent data from different objects (A, B, and C). In the tidy format, each column is a single variable, and each row is an observation. Note that the time data are repeated for each of the objects A, B, and C. The column id in the tidy data format identifies the condition. This column is automatically added and filled with a value of 1 when wide data are converted to tidy format. When multiple CSV files are uploaded with wide data, this column is filled with the filename.

For the wide format, the first column is assumed to contain the time points. Each of the other columns is taken as data of different samples. The wide format will be converted into the tidy format (Fig 1), and a column that identifies conditions, “id” is added. This conversion is not visible for the user, but it is required for plotting with ggplot2. When multiple wide data sets are simultaneously uploaded (in CSV or XLS format), each of these is treated as a different condition. This information is stored in the converted dataframe in the column id (Fig 1). A supplemental movie (S1 Movie) demonstrates how the multiple-file upload is done. The filename is used as a label for each condition.

### Tidy data format

For data in a tidy data format, the user is asked to select the columns that have (1) the time data, (2) the measured data (values), (3) a unique identifier for each sample, and (4) the condition (id). After data upload, there are several options to normalize the data to facilitate comparison. Some of the normalization options are divide values over a baseline value (I/I0), divide a difference by the baseline (ΔI/I0), and divide by the maximum value and scale between 0 and 1. The normalized data in a tidy format can be downloaded as a CSV file (comma separated values). Finally, there is an option to deselect data from individual objects.

## Data visualization

The default graph shows time along the x-axis and the measured values on the y-axis. A line plot is constructed by connecting all the coordinates from each sample by lines. The mean value and/or the 95% confidence interval of the mean can be drawn as a transparent layer on top of the lines from individual samples. The 95% confidence intervals enable the comparison of difference conditions by “visual inference” [16–18]. The line thickness and transparency of the individual data and statistical summary can be adjusted by the user. Several color palettes that are colorblindness friendly are available for the individual data and the statistics. When data for multiple conditions are provided, the statistics for each of the conditions can be shown with different colors. By tweaking the transparency, color, and line thickness, the display of the individual data and summary can be optimized.

## Display of data from individual objects

**Fig 2:**
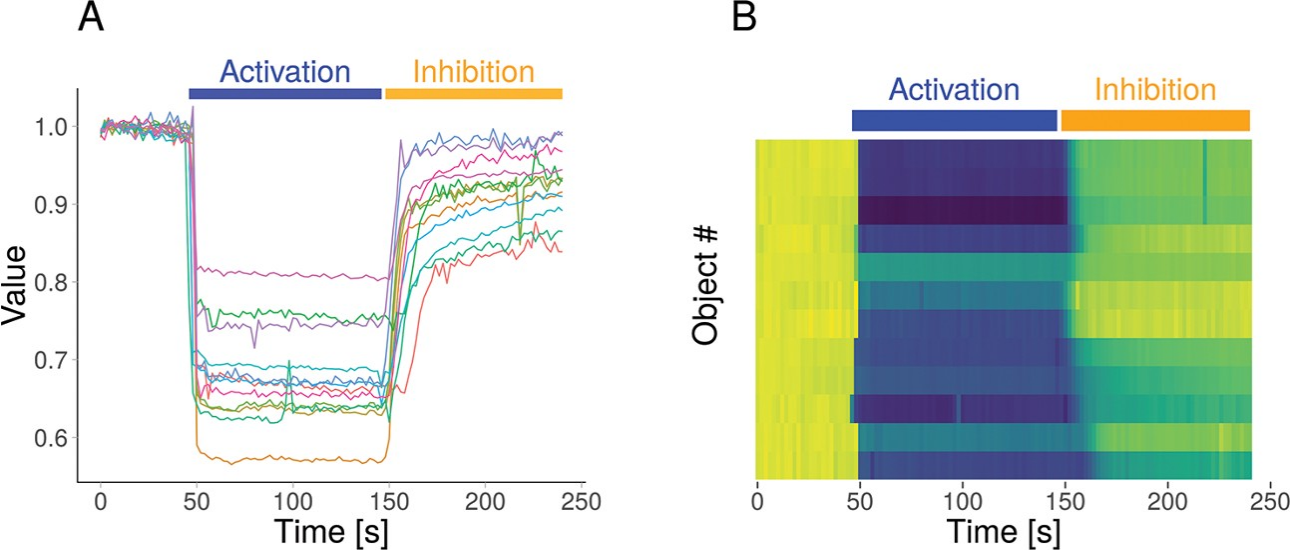
Different plots generated in PlotTwist with the same data. An ordinary line plot, showing the individual data as lines (A) and a heatmap-style presentation (B) of the (same) example data. Both graphs are annotated with bars on top of the plot to display an activating and an inhibiting perturbation.

When multiple objects (cells) are measured simultaneously, it is often useful to examine the responses of the individual objects. To this end, PlotTwist can show the data in 3 different ways: (1) a line plot, where single time-traces can be distinguished by color and/or labels; (2) small multiples showing each response in a separate graph; and (3) a heatmap-style representation that shows the response of each object in false color, also referred to as a lasagna plot [19]. The line plot is effective when the number of objects is low to medium (up to 20). The small multiples may still work well for up to 100 objects. The heatmap-style representation is especially suited for the display of rich data sets with many objects (>10 objects up to several hundreds). Fig 2 shows the same data in an ordinary line plot (Fig 2A) and in a heatmap-style presentation (Fig 2B). When a heatmap is used, the data from the different objects can be sorted according to several criteria, including the maximal signal, integrated response, and alphabetical order.

## Color

**Fig 3:**
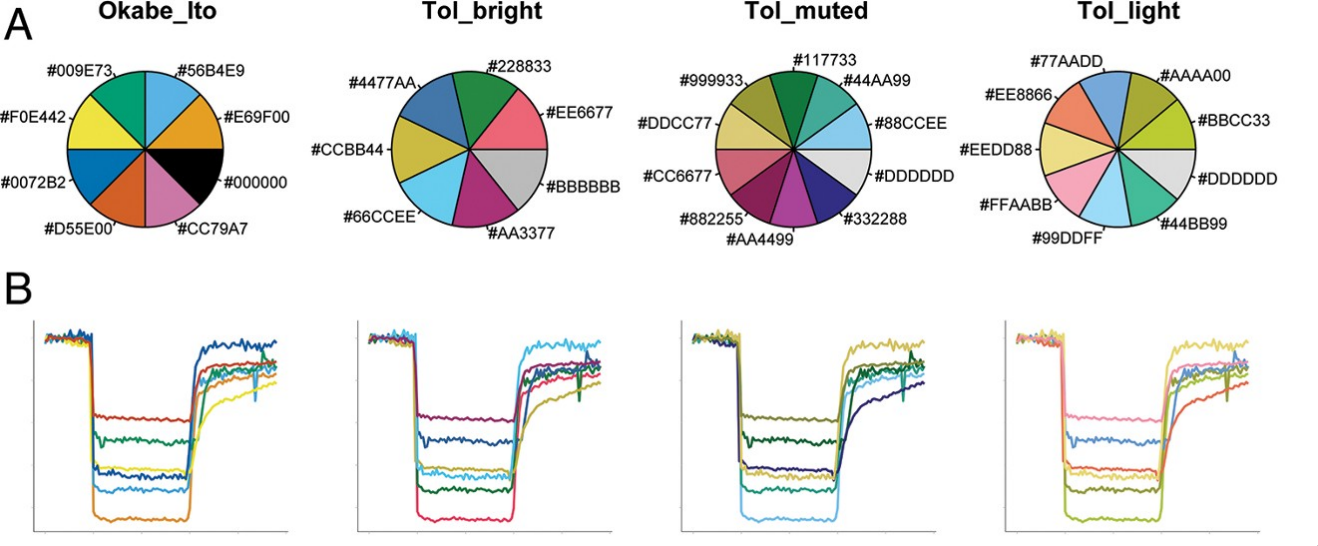
Composition and application of the color-blind-friendly palettes that are available in PlotTwist. (A) An overview of the color composition of the qualitative color palettes that were designed to have colors that are distinct to all people, including those with a color vision deficiency. The RGB code for each of the colors is indicated in hexadecimal code, preceded with a hashtag. (B) A realistic application of the color palettes shown in the upper panel for the unique labeling of 6 different lines. RGB, Red Green Blue.

Color is an important feature for distinguishing objects or conditions. To distinguish between different categories, qualitative color schemes are used. In PlotTwist, several qualitative color schemes are implemented that are color-blind friendly. The palettes have been designed by Masataka Okabe and Kei Ito (https://jfly.uni-koeln.de/color/) and Paul Tol (https://personal.sron.nl/~pault/). The palettes are composed of 7–10 colors, and their exact composition is shown in Fig 3A. The palette from Okabe and Ito is the default in PlotTwist. The most suitable color palette needs to be on a case-by-case basis. To give some idea about the performance of each of the palettes, we have used them to give unique colors to 6 different lines in a realistic plot (Fig 3B).

Factors that affect the choice of the palette may be the shape and thickness of the lines (thicker lines are usually easier to identify), their position and overlap, and the number of unique colors that are needed. In addition, the medium that is used to display the colors affects their appearance. The colors will look different on a screen, in print, or when projected with a beamer. It may be worthwhile to ask a person with a color vision deficiency which palettes work best, although color blindness is a heterogeneous deficiency [20].

Alternatively, user-defined colors can be applied by either typing the name or the hexadecimal Red Green Blue (RGB) color code—an example, also available in PlotTwist, of 3 different colors is “turquoise2, #FF2222, lawngreen,” in which #FF2222 is a hexadecimal RGB code for red. Whenever there are more objects than colors, the color scheme will be repeated, and therefore, different objects will be labeled with the same color.

For the heatmap-style presentation, the perceptually uniform and colorblindness-friendly viridis color palette is used (https://bids.github.io/colormap/).

## Labeling of data

**Fig 4:**
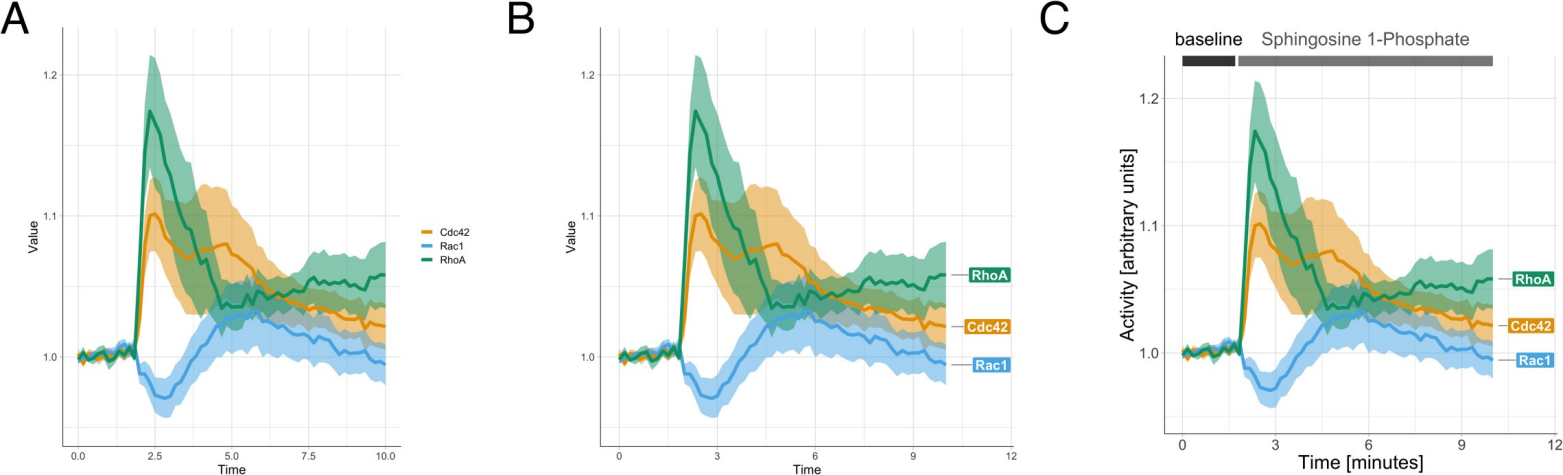
Different annotation styles that are available in PlotTwist. From left (A) to right (C), increasingly explicit labeling is used to explain the data. The data are the same for each plot and show the relative activity of the proteins Cdc42, Rac1, and RhoA in response to treatment with sphingosine 1-phosphate [14]. (A) A standard legend is used to explain the color used for the lines. (B) Direct annotation of the lines is used by placing a label next to the data. In addition, the color of the label is the same as the color of the data. (C) A fully annotated plot with clear labeling of the axes, direct annotation of the data, and a visualization of the treatments at the different times.

Colors can be supplemented with, or replaced by, direct labeling of the data. To this end, PlotTwist offers an option to add labels to the objects on the right side of the plot. The ggrepel package is used to reduce overlap between the labels. The labels show the name of the data. When only data are shown (no statistics), each individual line is labeled. In the case that the average curve is displayed, the label indicates the condition, next to the thick line that shows the average. When colors are used, the label has the same color as the data. Labels have the advantage that they (1) have a relatively large area filled with color, (2) indicate the condition with text in addition to color, and (3) are displayed next to the data. Therefore, labels can effectively replace legends. The use of labels instead of a standard legend is compared in Fig 4A and 4B.

## Indicating treatments

Time-lapse experiments are uniquely suited to analyze the effects of multiple treatments or perturbations during a single measurement. Some examples of perturbations in biological experiments are (1) the application of an agonist to stimulate signal transduction, (2) the addition of a drug to inhibit proteins or stimulate dimerization, or (3) illumination of light-sensitive proteins (optogenetics) to direct cellular processes. The annotation of plots to indicate the treatments is often done manually. This can be time consuming and inaccurate. To address this issue, PlotTwist enables straightforward and reproducible annotation of an unlimited number of treatments. The annotation can be added as a bar above the plot, a box in the plot, or a combination of both. The color of the annotation can be adjusted, and text can be added. An example of annotations is shown in Figs 2A, 2B and 4C, where the treatments are indicated by bars on top of the plot. Next to bars, there is also an option to use boxes or a combination of a bar and box.

## Output

The data that are used for the plot can be downloaded in a CSV format in the tidy format. Any deselected data will not appear in the file. In the case that the data were subjected to normalization, there is also an option to download these data as a CSV file in tidy format. Downloaded CSV files can be used again by PlotTwist to plot the data.

The plot that is generated by the app can be directly retrieved by drag-and-drop from the web browser. In addition, the plot can be downloaded as a portable network graphics (PNG) or portable document format (PDF) file. The PNG is a lossless bitmap format. The PDF allows for downstream processing/editing with software that can handle vector-based graphics.

## Reproducibility

In general, graphical user interfaces offer substantial flexibility in the options and parameters that can be set. In PlotTwist, the user has control over scaling, annotation, color, and more. Because of this flexibility, it is challenging to exactly reproduce the settings. Here, to achieve reproducibility, we have included an option to “clone” the active setting. This action will generate a uniform resource locator (URL) with all relevant parameters from the user interface. Through the URL, the parameters are fed back into the web tool and the settings are restored. As an example, Fig 2A can be reproduced by employing this URL:

https://huygens.science.uva.nl/PlotTwist/?data=1;;;fold;1,5;&vis=dataasline;1;;;1;&layout=%20;TRUE;;%20;;TRUE;;1;X;480;480&color=none&label=TRUE;A;TRUE;Time%20[s];Value;TRUE;30;24;18;8;;&stim=TRUE;bar;46,146,148,240;Activation,Inhibition;Blue,Orange&.

The plot in Fig 4C can be recreated by using this link:

https://huygens.science.uva.nl/PlotTwist/?data=2;TRUE;;fold;1,5;&vis=dataasline;0;TRUE;TRUE;1;;&layout=;;TRUE;;0.95,1.22;TRUE;TRUE;6;X;600;600&color=none&label=;;TRUE;Time [minutes];Activity [arbitrary units];TRUE;24;24;18;8;;;TRUE&stim=TRUE;bar;0,1.7,1.

In supplemental text (S1 Text), all the variables (and their accepted values) that can be set through the URL are listed. The access to the variables (S1 Text) enables users to make presets. For instance, when a certain set of graphs need to be annotated in the same way, this can be achieved by passing the relevant parameters through the URL. For annotation with 3 labels (baseline, activation, inhibition) at times 10–40, 60–140, and 170–240, the following URL can be used:

https://huygens.science.uva.nl/PlotTwist/?stim=TRUE;bar;10,40,60,140,170,240;baseline,activation,inhibition.

Another example for setting the default to remove the grid and use thick lines and color for the data is as follows:

https://huygens.science.uva.nl/PlotTwist/?layout=;T;;;;T&vis=;1;;;;;T.

After using the URL to start PlotTwist, the data can be uploaded, and the (preset) annotation will be present in the plot that visualizes the imported data. Another way to generate presets is by cloning the URL for a setting that is right and copying the variables that are of interest.

## Clustering

Clustering is an unsupervised method for grouping data based on similarity, and it is used to reveal patterns in data. Clustering has been used on high-content time-lapse data acquired from single cells [21–23]. To facilitate cluster analysis, several methods are implemented in PlotTwist. Because the application of clustering on time-series data is relatively new, it is unclear which clustering method gives the best biological insight. Another aspect of cluster analysis is to find the right number of clusters. To provide guidance as to the clustering method and cluster number, a cluster validation index (CVI) can be calculated [13]. Three different CVIs are implemented that return a number for a specific number of clusters. The higher the number, the better the clustering. It should be noted that the CVI is a statistic that summarizes the data and should be treated accordingly. As such, the number of clusters that is optimal according to the CVI does not necessarily reflect the underlying biology. PlotTwist enables the rapid evaluation of different clustering methods and their quality. More options for inspecting time series and assessing cluster quality are available in the “Time Course Inspector” application generated by Dobrzyński and colleagues [24].

**Fig 5:**
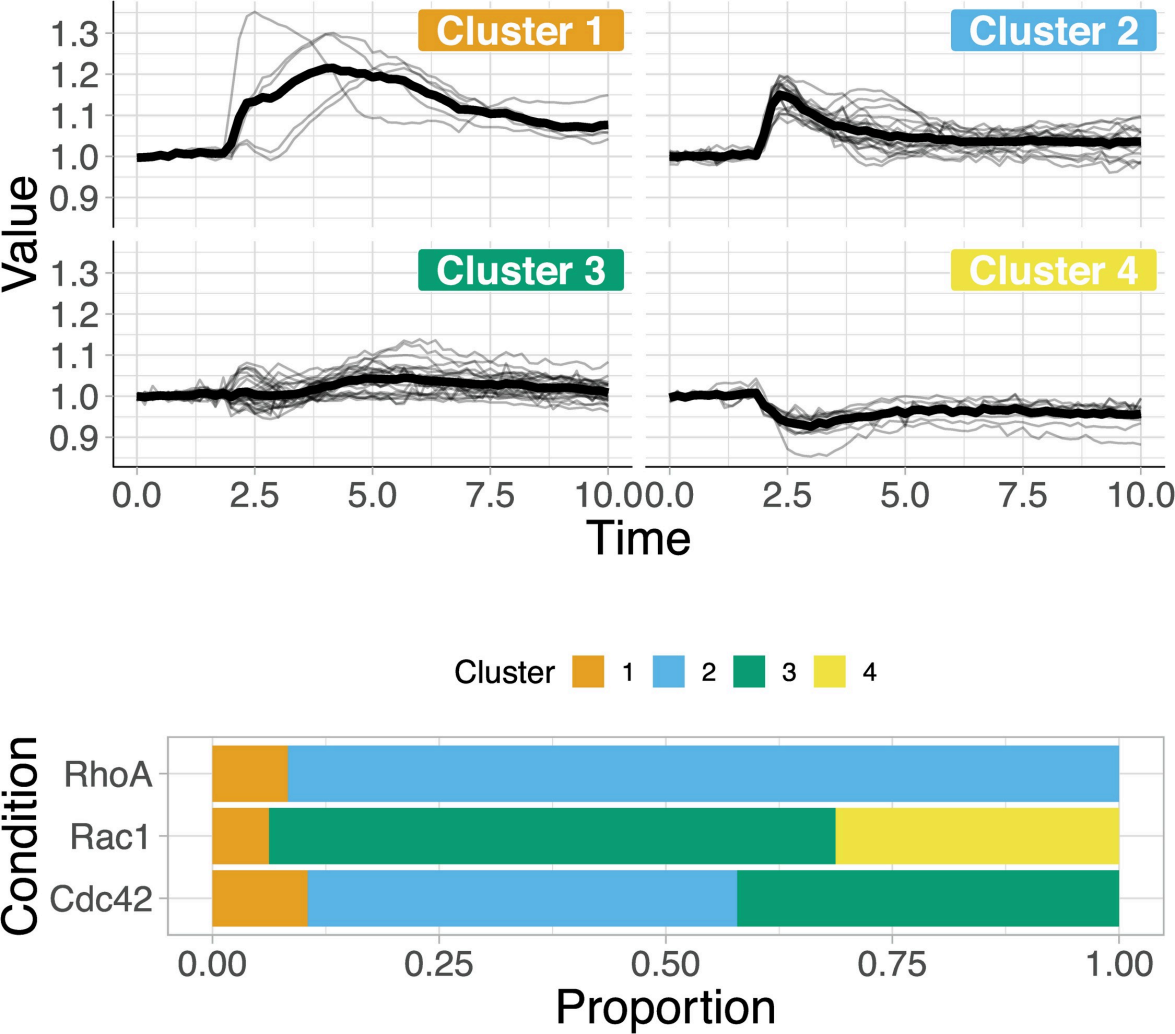
Result of the clustering of data set Example 2 in PlotTwist. The data set was subjected to clustering with Euclidean distance and Ward.D2 linkage, using an optimal number of 4 clusters as inferred from the Calinski Harabasz index. The plots show the data allocated to a cluster and how the data of the 3 Rho GTPases (RhoA, Rac, and Cdc42) are distributed over the 4 different clusters.

To illustrate clustering, the data that were previously acquired for the response of 3 Rho GTPases [25] were used (example data 2 in the PlotTwist app). Clustering of the entire data set based on Euclidean distance returned a maximal value for the Calinski Harabasz index of 45.76 for 4 clusters. The time series are displayed according to the clusters (Fig 5), showing similarity within a cluster but differences between clusters. The RhoA dynamics are mainly represented by cluster 2, whereas Rac1 dynamics fall in clusters 3 and 4, demonstrating strikingly different signaling dynamics between these Rho GTPases. To conclude, the option of clustering in PlotTwist may provide an unbiased way for finding similarities in continuous data.

## Conclusion

Performing measurements over time (or any another continuous parameter) on several objects is an important experimental strategy. The resulting data can be high content (when many objects are measured) and complex (due to application of different treatments or perturbations). To evaluate the results, data visualization tools that can rapidly switch between different presentation modes are needed. To communicate the results, state-of-the-art data visualization tools that enable clear annotations are needed. We have developed the freely available and open-source tool PlotTwist to serve both purposes. It can be used for inspection of data and rapidly switch between different presentations, i.e., standard line plot, small multiples, and heatmap. Basic statistics (mean and 95% confidence interval) can be added in user-defined transparency and color. To generate publication-ready figures, we use the state-of-the-art graphical package (ggplot2) to make the plots. The flexible and reproducible annotation of both data and experimental conditions allows for clear communication of the results. In summary, PlotTwist allows anyone to inspect data and to generate state-of-the art data visualizations. Although this application was written with time-dependent biological processes in mind, it could be of use for any other data set with continuous data.

## Supporting information

S1 Text Passing parameters to PlotTwist through the HTML address. (DOCX)

S1 Movie Demonstration of multiple-file upload to plot data for different conditions. (MOV)

## Abbreviations

CVI: cluster validation index
CSV: comma-separated values
PDF: Portable Document Format
PNG: Portable Network Graphics
RGB: Red Green Blue
URL: uniform resource locator
XLS: eXceL Spreadsheet
